# Is testicular microlithiasis associated with decreased semen parameters? a systematic review

**DOI:** 10.1186/s12610-024-00238-x

**Published:** 2024-12-05

**Authors:** Hannah G. Wilson, Brian R. Birch, Rowland W. Rees

**Affiliations:** 1 Faculty of Medicine, University of Southampton, University Road, Southampton, Hampshire SO17 1BJ UK; 2 Department of Urology, University Hospital Southampton NHS Foundation Trust, Tremona Road, Southampton, Hampshire SO16 6YD UK

**Keywords:** Testicular Microlithiasis, Semen Parameters, Sperm Concentration, Sperm Motility, Sperm Morphology, Microlithiase testiculaire, Paramètres du Sperme, Concentration des Spermatozoïdes, Mobilité des Spermatozoïdes, Morphologie des Spermatozoïdes

## Abstract

**Background:**

Testicular microlithiasis (TM) is characterised by microcalcifications in the testes and has been associated with infertility. This has led to studies of semen analysis in men with the condition. This systematic review aimed to compare semen parameters in men with TM and those without. Men with classic TM (≥ 5 microcalcifications per sonographic image) were also compared to those with limited TM (< 5 microcalcifications per sonographic image). Additionally, testicular volume and hormone levels were analysed as secondary outcomes. This review was carried out according to PRISMA guidelines and registered on PROSPERO. The quality of included studies was assessed using the Newcastle–Ottawa Scale.

**Results:**

Embase, MEDLINE, World of Science and Scopus were searched. Abstracts were screened against inclusion/exclusion criteria by two independent reviewers. Eligible studies included data on semen parameters in men with TM where semen analysis was done according to World Health Organisation recommendations. Studies with populations consisting of men with testicular cancer were excluded. After searching the databases, 137 papers were found and 10 studies involving 611 men with TM were included in the analysis. In the studies that compared sperm concentration in men with TM to controls, six (100%) found lower sperm concentration in the TM group. Six studies compared sperm motility, of which 4 (66.7%) showed lower motility in the TM group compared to controls. Five studies compared sperm morphology, with three (60%) finding a lower percentage of normal morphology in the TM group compared to controls. Six studies compared classic TM with limited TM. All six (100%) found a lower sperm concentration in the classic TM group compared to the limited TM group. Results also suggested that more extensive disease is associated with poorer sperm concentration.

**Conclusions:**

This review suggests that TM is associated with decreased semen parameters, particularly sperm concentration. However, clinical outcomes should be investigated by studying pregnancy rates in males with TM. Future research that controls for confounding variables, involves larger sample sizes, and utilises advanced sperm function tests is also advised. Further research is important for establishing clinical guidance and suggestions for fertility follow-up in men with TM.

**Supplementary Information:**

The online version contains supplementary material available at 10.1186/s12610-024-00238-x.

## Résumé

**Contexte:**

La microlithiase testiculaire (MT) est caractérisée par des microcalcifications dans les testicules, et a été associée à l’infertilité. Ceci a conduit à des études sur l’analyse du sperme chez les hommes atteints de cette maladie. Cette revue systématique visait à comparer les paramètres du sperme chez les hommes atteints de MT et ceux qui non atteints. Les hommes atteints de MT classique (≥ 5 microcalcifications par image échographique) ont également été comparés à ceux qui présentaient une MT limitée (< 5 microcalcifications par image échographique). De plus, le volume testiculaire et les taux d’hormones ont été analysés comme critères de jugement secondaires. Cette étude a été réalisée conformément aux directives PRISMA et enregistré sur PROSPERO. La qualité des études incluses a été évaluée à l’aide de l’échelle de Newcastle-Ottawa.

**Résultats:**

Les recherches ont été menées sur Embase, MEDLINE, World of Science et Scopus. Les résumés ont été examinés en fonction des critères d’inclusion et d’exclusion par deux examinateurs indépendants. Les études éligibles comprenaient des données sur les paramètres du sperme chez les hommes atteints de MT chez lesquels l’analyse du sperme avait été effectuée conformément aux recommandations de l’Organisation mondiale de la santé. Les études portant sur des populations composées d’hommes atteints d’un cancer des testicules ont été exclues. Après consultation des bases de données, 137 articles ont été trouvés et 10 études impliquant 611 hommes atteints de MT ont été incluses dans l’analyse. Dans les études qui ont comparé la concentration de spermatozoïdes chez les hommes atteints de MT à celle des témoins, six (100%) ont trouvé une concentration de spermatozoïdes plus faible dans le groupe MT. Six études ont comparé la mobilité des spermatozoïdes, dont 4 (66,7%) ont montré une mobilité plus faible dans le groupe MT par rapport aux témoins. Cinq études ont comparé la morphologie des spermatozoïdes, trois (60%) ont trouvé un pourcentage plus faible de morphologie normale dans le groupe MT par rapport aux témoins. Six études ont comparé la MT classique à la MT limitée. Les six (100%) ont trouvé une concentration de spermatozoïdes plus faible dans le groupe de MT classique par rapport au groupe de MT limitée. Les résultats ont également suggéré qu’une maladie plus étendue est associée à une plus faible concentration de spermatozoïdes.

**Conclusions:**

Cette revue suggère que la MT est associée à une diminution des paramètres du sperme, en particulier la concentration des spermatozoïdes. Cependant, les résultats cliniques devraient être étudiés en prenant en compte les taux de grossesse chez les hommes atteints de MT. Des recherches futures avec contrôle des facteurs de confusion, impliquant des échantillons de plus grande taille et utilisant des tests avancés de la fonction spermatique sont également conseillées. Des recherches supplémentaires sont nécessaires afin d’établir des recommandations cliniques et des suggestions pour le suivi de la fertilité chez les hommes atteints de MT.

## Introduction

**Fig. 1:**
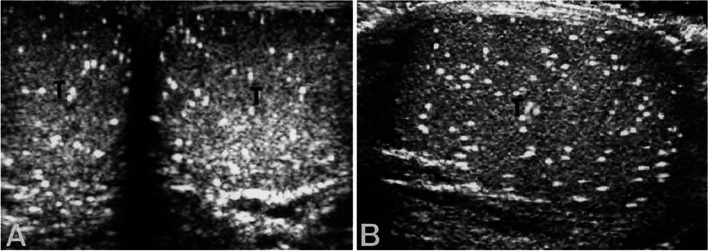
Ultrasound Scan of Classic Testicular Microlithiasis. Ultrasound Scan of Classic Testicular Microlithiasis in a 35-year-old male. **A**: both testes, transverse view. **B**: right testis, longitudinal view. Image by Kim et al. [6] used with permission

Testicular Microlithiasis (TM) is a condition that is characterised by the presence of microcalcifications in the testes [1]. These microcalcifications can range from 1-3 mm [1] and generally have a diffuse and symmetrical distribution, although there can be variation [2–4]. TM is usually found incidentally by ultrasound (US) [3] (Fig. 1A, B) and the sonographic appearance was first described by Doherty et al. [5] in 1987 as ‘a pattern of innumerable tiny bright echoes’. Since then, the introduction of higher frequency US has led to more cases of TM being reported [6].

Definitions of TM vary subtly in the literature [1, 7, 8] and studies investigating TM may categorise microcalcifications into classic testicular microlithiasis (CTM), ≥ 5 microcalcifications per sonographic image, and limited testicular microlithiasis (LTM), < 5 microcalcifications per sonographic image [9–11]. TM may also be visualised histologically as deposits of laminated calcifications or haematoxylin bodies, with a study of Danish and English men finding different proportions of the histopathological types in these populations [12]. These calcifications are composed of hydroxyapatite and are found in the seminiferous tubules [13, 14]. However, some researchers state that the microliths are extratubular in origin [15]. The aetiology of TM remains uncertain although several causative mechanisms have been suggested including Sertoli cell dysfunction [15, 16], abnormal gonadal embryogenesis [15], nanobacteria [17] and trauma [18, 19]. Furthermore, there may be a genetic basis in some individuals [15, 20, 21]. Interestingly, studies have also found that black men have a higher prevalence of the condition, indicating associations between ethnicity and TM [22, 23].

TM has been associated with infertility, with the prevalence of TM in subfertile and infertile populations of males ranging between 0.8% [24] and 20% [25]. TM has been proposed to cause infertility by several mechanisms and studies have shown that microcalcifications may occupy 30% to 60% of seminiferous tubules [26, 27]. However, the patients included in these studies had cryptorchidism which could be a confounding factor. Smith et al. [28] found that blockage of seminiferous tubules due to microliths led to a build-up of cellular debris, and sperm isolated from affected testes had higher levels of abnormalities. Obstruction of the seminiferous tubules may also cause inflammation, increased intra-seminiferous pressure and have an impact on the blood supply of the testes [17]. All of these factors could affect spermatogenesis however more contemporary research is needed. There is currently no definitive causative mechanism that links TM to infertility.

Although semen analysis alone cannot indicate fertility status, the association between TM and infertility has led to studies focused on the comparison of semen parameters in men with TM to those without. Some studies have found no difference in males with TM compared to normal controls [29, 30] however, a case–control study by Mahafza et al. [31] found statistically significant differences in multiple semen parameters when those with TM were compared to those without. Notably, lower sperm concentration, motility and normal morphology were found in the TM group in comparison with control subjects. A 2020 study by Rassam et al. [32] supports these findings with sperm concentration, morphology, and motility being significantly lower in males with microcalcifications compared to a control group without. However, although Rassam et al. [32] found microliths to be an indicator of poorer sperm quality, they did not find a significant difference between semen parameters in males with CTM compared to LTM. Other studies report contrary findings. A 2020 study by Hiramatsu et al. [33] found that sperm concentration correlated negatively with the number of microcalcifications present and multiple other studies [9, 34, 35] support this, with semen parameters found to be significantly worse in those with CTM compared to individuals with LTM.

Other measurements such as hormone concentrations and testicular volume are also commonly reported in the literature alongside semen analysis results. Increased FSH and reduced testicular volume are indicators of germinal epithelial damage and are associated with low sperm count in infertile men [18]. Considering FSH, significantly higher levels were found in CTM groups [9, 35] although total testosterone levels have not been found to be significantly different between CTM and LTM patients [9]. D’andrea et al. [9] found that CTM was associated with lower testicular volume, with other studies [35] also supporting this finding.

In summary, there are contradictory findings in the literature regarding TM and semen parameters. Given this gap in the literature, this systematic review aimed to investigate the association of TM with decreased semen parameters. The primary aim was to compare sperm concentration, morphology, and motility in men with TM to those defined as not having TM. Secondary aims included investigating semen parameters in men with CTM compared to LTM as well as collecting data related to testicular volume and hormone levels (where available).

## Materials and methods

The review protocol was registered on the “International Prospective Register of Systematic Reviews” (PROSPERO), PROSPERO ID: CRD42022368857 [36]. The review was carried out using the “Preferred Reporting for Systematic Reviews and Meta Analyses” (PRISMA) [37] recommendations.

### Search strategy

The following databases were searched on 4th October 2022: (Ovid) Embase classic + Embase 1947 to 2022 week 39, (Ovid) MEDLINE(R) ALL 1946 to October 03 2022, (Clarivate Analytics) Web of Science Core Collection and Scopus. No date, language or publication type restrictions were enforced at this stage. Individual search strategies were created for different databases to accommodate different medical subject headings (MeSH) and command operators. In general, the wildcard testic* and the operator ADJ3 (microlithiasis OR calcification OR microcalcification) were combined by the Boolean operator AND with (semen OR sperm OR seminal) ADJ3 (count OR number OR motility OR morphology OR concentration OR volume OR parameters OR quality). Full search strategies for each database can be seen in Additional File 1. OpenGrey and Mednar were used to search the grey literature to provide enhanced subject coverage. Citation searching was also carried out in papers included after full-text screening to identify any missing literature. The original search strategies were re-run on 22nd April 2024 to ensure that no additional studies had been published in the interim.

### Eligibility criteria

Studies in any language were considered eligible if they included men with TM (which could be documented as TM, CTM or LTM) and if the reported outcomes included semen parameters which were analysed according to World Health Organisation (WHO) guidelines [38, 39]. Due to inconsistencies in definitions used in studies, TM was classified as the presence of microcalcifications in the testes. CTM was defined as ≥ 5 microcalcifications per sonographic image and LTM as < 5 microcalcifications per sonographic image. This criterion was included as CTM and LTM are commonly reported distinctions in studies that investigate semen parameters in men with TM.

Studies were excluded if they had non-human participants, were review articles or case reports, or had population crossover. Where papers had population crossover (either the same population reported in different papers or an overlapping population in different papers) the most appropriate paper concerning the review question was included. This was done to avoid overstating results derived from the same cohort of patients [40]. Studies where the participants consisted of testicular cancer patients were also excluded as orchidectomy and other treatments given to this group could impact semen parameters.

### Data extraction

References and abstracts of papers found from the database search were exported to Endnote [41] where duplications were removed by automation and manual search. The remaining papers were exported to Rayyan [42] where the abstracts were screened against the eligibility criteria by both reviewers HW and RR. Papers which fit the eligibility criteria were then reviewed as full-text papers by HW and RR. The following data was then extracted from papers that were included in the review: Publication data (title, authors, year of publication), Study characteristics (study design, number of participants, how the population was selected) and numerical values of the outcome measures (sperm concentration, sperm morphology, sperm motility and other parameters including sperm count and semen volume) as well as associated *p* values. A reported *p* < 0.05 2-tailed was deemed statistically significant. Additional participant characteristics such as testicular volume and hormone levels were also extracted. Data was recorded electronically in Microsoft Excel and the data table was piloted prior to data extraction to assess suitability.

Due to heterogeneity in the way results were reported between studies and study designs, meta-analysis and a funnel plot to assess publication bias could not be carried out. Authors of included studies were also contacted for the raw data needed for statistical analysis however no responses were received. Narrative synthesis was the primary method of analysis.

### Additional calculations

Some studies [9, 34] had data that required further mathematical manipulation to render the review outcomes. To convert results reported as medians and interquartile ranges into means and standard deviations (SD), formulae from papers by Luo et al. [43] and Wan et al. [44] were used. If groups had skewed data rather than a normal distribution these calculations were not applied. To combine the means and SDs of two groups (for example combining data from a CTM and LTM group to find a value for the TM group as a whole) the Cochrane formulae [45] were used. The study by Xu et al. [35] did not state whether the SD or standard error was used and therefore calculations could not be applied to combine the data from the CTM and LTM groups. The authors of the study were contacted but no response was received. Where studies presented individual patient data, the data was combined to calculate the mean and SD. If not reported, *p* values were calculated from means, SDs, and sample sizes.

The research study conducted by Xu et al. [35] did not include a *p*-value for the difference between the TM and non-TM groups and it was not possible to calculate a *p*-value from the data provided. However, an ANOVA of 3 groups (TM, CTM, and LTM) showed a *p*-value of < 0.001 for both sperm concentration and sperm motility. The comparison between CTM and non-TM groups also had a *p*-value of < 0.001 for these parameters. Based on these highly significant *p* values and the numerical difference between the mean values from the TM and non-TM groups, the decrease in the TM group in comparison to the non-TM group was assumed to be statistically significant for sperm concentration and sperm motility for the purpose of comparison in Table 1. This analysis was done with formal statistical input.

**Table 1:** Comparison of semen parameters (sperm concentration, morphology and motility) in 10 studies that contained data of semen parameters in men with testicular microlithiasis

**Authors and reference number**	**Year**	**Number in Sample**		**Sperm Concentration (million/ml)**			**Sperm Morphology** (**% normal sperm morphology)**			**Sperm motility (Total motility, PR + NP (%) or PR (%))**		
		TM	Control	Lower concentration compared to control group?	Statistically significant difference?	Is TM group on or below WHO 2021 decision limit?	Lower normal morphology compared to control group?	Statistically significant decrease?	Is TM group on or below WHO 2021 decision limit?	Lower motility compared to control group?	Statistically significant difference?	Is TM group on or below WHO 2021 decision limit?
***Infertile Population:***												
D’Andrea et al. [9]	2021	76	81				**✓**	**✗**	**✗**			
Jiang et al. [48]	2013	22	0			✓			✓			✗
Ou et al. [10]	2007	23	0			**✗**			**✗**			**✗**
Rassam et al. [32]	2020	218	2696	✓	✓	✓	✗	✗	✗	✓	✓	✗
Sakamoto et al. [49]	2006	31	519	✓	✗	✗				✗	✗	✓
Thomas et al. [34]	2000	10	0			**✗**						**✗**
Yee et al. [50]	2011	10	50	✓	✗	✗	✗	✗	✗	✓	✗	✓
***Asymptomatic/healthy population:***												
Anvari Aria et al. [51]	2020	42	4797	✓^a^	✓^a^	✗	✓	✗	✗	✗	✗	✗
***Population status not specified:***												
Mahafza et al. [31]	2016	20	23	✓	✓	✗	✓	✓	✗	✓	✓	✗
Xu et al. [35]	2014	159	120	**✓**	**✓**	**✗**				**✓**	**✓**	**✗**

Summary table of the included studies that contained data on sperm concentration, morphology and motility in men with testicular microlithiasis. The table indicates whether the testicular microlithiasis group had lower semen parameter values compared to the control group, if these differences were statistically significant and compares the semen parameter values to the 2021 World Health Organisation decision limits. *TM* testicular microlithiasis, *WHO* World Health Organisation, *PR* progressively motile, *NP* non-progressively motile, ✓ Yes (in response to the question in the respective column), ✗ No (in response to the question in the respective column), a: bilateral TM only. Bold indicates that values have been calculated by the review authors from data in the study (where applicable, the statistical test used was the independent samples t-test). Blank spaces indicate that there was insufficient data available to answer the question

### Quality assessment

Quality assessment of included studies was done using the Newcastle–Ottawa scale (NOS) for case–control studies [46] and the Newcastle–Ottawa Scale adapted for cross-sectional studies by Herzog et al. [47]. The NOS appraises different domains such as selection, comparability and either outcome or exposure to aid in the quality assessment of studies. The NOS for case–control studies awards a maximum of 9 points per study and the NOS adapted for cross-sectional studies awards a maximum of 10 points per study. Once all domains were assessed the following scoring ranges were used: 0–3 = low quality, 4–6 = medium quality, 7–9/10 = high quality. The scales specific to this review can be viewed in Additional File 2.

## Results

### Study selection

**Fig. 2:**
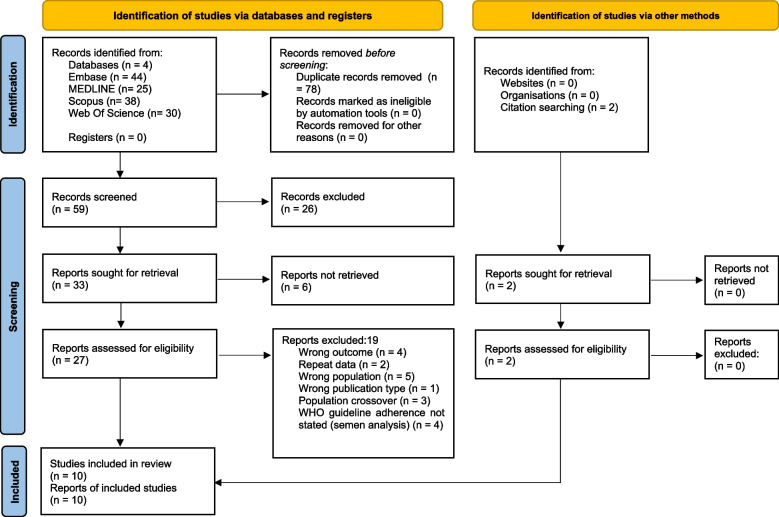
“Preferred Reporting Items for Systematic reviews and Meta-Analyses” (PRISMA) flow diagram detailing the inclusion and exclusion of papers. Flow diagram detailing how searching 4 databases led to 10 papers being included in this review. First duplicates were removed, then titles and abstracts were screened. This was followed by full-text screening. Citation searching was also carried out. This process was carried out by 2 independent reviewers

Searching the 4 databases led to 137 papers being located. After deduplication 59 remained. 63 duplicates were found automatically on endnote and 15 were found manually. Screening of titles and abstracts resulted in 33 papers being included for full-text screening. After full text screening 8 remained. 2 further papers were found by citation searching leading to 10 papers being included in the review. Figure 2 adapted from the PRISMA 2020 statement [37] shows the process of inclusion and exclusion. Additional File 3 details reasons of exclusion from full text screening.

### Study characteristics

The 10 studies included all had data on semen parameters in participants with TM, some also included data on hormone levels as well as testicular volume. Altogether there were 8897 participants included in the 10 studies who contributed to semen parameter data, of which 611 were classified as having TM. 214 participants with CTM were also compared to 261 participants with LTM.

Seven (70.0%) studies [9, 10, 32, 34, 48–50] included males from an infertile population. One (10.0%) study [51] included males from an asymptomatic/healthy population and 2 (20.0%) studies [31, 35] included males from unspecified populations. These distinctions are included in the results tables.

Eight (80.0%) studies [10, 32, 34, 35, 48–51] had a cross-sectional design whilst 2 (20.0%) studies [9, 31] were case–control studies.

Two studies [35, 48] were based in China, and the rest in the following countries: Italy [9], Japan [49], Taiwan [10], Germany [32], South Korea [50], United Kingdom [34], Denmark [51] and Jordan [31]. Due to differences in reporting, the mean age of participants across all studies could not be calculated. However, data for the age of participants in each study can be seen in Additional File 5.

Nine (90.0%) studies [9, 10, 31, 34, 35, 48–51] were rated as medium quality by the NOS (scores of 4–6) and 1 (10.0%) study [32] was scored as being high quality (score of 7–9/10). The full quality assessment table of results can be seen in Additional File 4.

### Results of included studies

Additional File 5 includes the full results and numerical data from all 10 studies included in the systematic review, including data on sperm count and semen volume which were inconsistently reported among studies. Papers differed in the parameters they reported and how they reported outcomes therefore, not all studies had sufficient data to answer all the outcomes of this review.

Table 1 summarises the results of 10 studies that recorded values for sperm concentration, morphology or motility (or that could have these values calculated from data reported) in participants with TM.

Table 2 summarises the results of 6 studies that compared values for sperm parameters in males with CTM compared to those with LTM.

**Table 2:** Comparison of semen parameters (sperm concentration, morphology and motility) in 6 studies that compared men with classic testicular microlithiasis (≥ 5 microcalcifications per sonographic image) to those with limited testicular microlithiasis (< 5 microcalcifications per sonographic image)

**Authors and reference number**	**Year**	**Number in Sample**		**Sperm Concentration (million/ml)**			**Sperm Morphology** (**% normal sperm morphology)**			**Sperm motility (Total motility, PR + NP (%) or PR (%))**		
		CTM	LTM	Lower concentration compared to LTM group?	Statistically significant difference?	Is CTM group on or below WHO decision limit?	Lower normal morphology compared to LTM group?	Statistically significant difference?	Is CTM group on or below WHO decision limit?	Lower motility compared to LTM group?	Statistically significant difference?	Is CTM group on or below WHO decision limit?
***Infertile Population:***												
D’Andrea et al. [9]	2021	34	42	✓	✓	✓	✗		✗	✗		✗
Ou et al. [10]	2007	11	12	✓	✗	✗	✗	✓^a^	✗	✗	✓^a^	✗
Rassam et al. [42]	2020	53	134	✓	✗	✓	✗	✗	✓	✓	✗	✓
Thomas et al. [34]	2000	5	5	**✓**	**✗**	**✓**				**✓**	**✓**	**✓**
***Population status not specified:***												
Mahafza et al. [31]	2016	14	6	✓	✗	✗	✓	✗	✗	✗	✗	✓
Xu et al. [35]	2014	97	62	✓	✓	✗				✓	✓	✗

Summary table of the included studies that compared sperm concentration, morphology and motility in men with classic testicular microlithiasis to those with limited testicular microlithiasis. The table indicates whether the classic testicular microlithiasis group had lower semen parameter values compared to those with limited testicular microlithiasis, if these differences were statistically significant and compares the semen parameter values to the 2021 World Health Organisation decision limits. *CTM* classic testicular microlithiasis, *LTM* limited testicular microlithiasis, *WHO* World Health Organisation, *PR* progressively motile, *NP* non-progressively motile, ✓ Yes (in response to the question in the respective column), ✗ No (in response to the question in the respective column), a: CTM group had significantly higher results compared to LTM group. Bold indicates that values have been calculated by the review authors from data in the study. Blank spaces indicate that there was insufficient data available to answer the question

Tables 3 and 4 summarise findings for hormone levels and Tables 5 and 6 summarise findings for testicular volume.

**Table 3:** Comparison of FSH, LH and testosterone levels in 4 studies that compared men with testicular microlithiasis to those in control groups

Authors and reference number	Year	Number in Sample		FSH		LH		Testosterone	
		TM	Control	Difference compared to control group?	Statistically significant difference?	Difference compared to control group?	Statistically significant difference?	Difference compared to control group?	Statistically significant difference?
***Infertile Population:***									
D’Andrea et al. [9]	2021	93	97					**↓**	**✗**
Rassam et al. [32]	2020	218	2696	↑	✓	↑	✗	↓	✗
Sakamoto et al. [49]	2006	31	519	↓	✗	↑	✗		
***Asymptomatic/healthy population:***									
Anvari Aria et al. [51]	2020	42	4797	↑^a^	✗	↓	✗	↑	✗

Summary table of the included studies that compared follicle stimulating hormone, luteinising hormone and testosterone levels in men with testicular microlithiasis compared to controls. The table indicates the direction of the difference in the testicular microlithiasis group compared to the control group and if the difference was statistically significant. *TM* testicular microlithiasis, *FSH* follicle stimulating hormone, *LH* luteinising hormone, ✓ Yes (in response to the question in the respective column), ✗ No (in response to the question in the respective column), ↓: decrease, ↑: increase, a: bilateral TM only. Bold indicates that values have been calculated by the review authors from data in the study (where applicable, the statistical test used was the independent samples t-test). Blank spaces indicate that there was insufficient data available to answer the question

**Table 4:** Comparison of FSH, LH and testosterone levels in 3 studies that compared men with classic testicular microlithiasis (≥ 5 microcalcifications per sonographic image) to those with limited testicular microlithiasis (< 5 microcalcifications per sonographic image)

Authors and reference number	Year	Number in Sample		FSH		LH		Testosterone	
		CTM	LTM	Difference compared to LTM group?	Statistically significant difference?	Difference compared to LTM group?	Statistically significant difference?	Difference compared to LTM group?	Statistically significant difference?
***Infertile Population:***									
D’Andrea et al. [9]	2021	46	47	↑	✓	↑		↓	**✗**
Rassam et al. [32]	2020	53	134	↑	✗	↓	✗	↓	✗
***Population status not specified:***									
Xu et al. [35]	2014	97	62	↑	✓	↑		↓	

Summary table of the included studies that compared follicle stimulating hormone, luteinising hormone and testosterone levels in men with classic testicular microlithiasis compared to men with limited testicular microlithiasis. The table indicates the direction of the difference in the classic testicular microlithiasis group compared to the limited testicular microlithiasis group and if the difference was statistically significant. *CTM* classic testicular microlithiasis, *LTM* limited testicular microlithiasis. *FSH* follicle stimulating hormone, *LH* luteinising hormone ✓ Yes (in response to the question in the respective column), ✗ No (in response to the question in the respective column), ↓: decrease, ↑: increase. Bold indicates that values have been calculated by the review authors from data in the study (where applicable, the statistical test used was the independent samples t-test). Blank spaces indicate that there was insufficient data available to answer the question

**Table 5:** Comparison of average testicular volume in 4 studies that compared men with testicular microlithiasis to those in control groups

Authors and reference number	Year	Number in Sample		Testicular volume	
		TM	Control	Volume compared to control	Statistically significant difference?
***Infertile Population:***					
D’Andrea et al. [9]	2021	93	97	**↓**	**✗**
Rassam et al. [32]	2020	218	2696	↓	✗
Sakamoto et al. [49]	2006	31	519	↓	✗
***Asymptomatic/healthy population:***					
Anvari Aria et al. [51]	2020	42	4797	↓	

Summary table of the included studies that compared testicular volume in men with testicular microlithiasis compared to controls. The table indicates the direction of the difference in the testicular microlithiasis group compared to the control group and if the difference was statistically significant. *TM* testicular microlithiasis ✓: Yes (in response to the question in the respective column), ✗: No (in response to the question in the respective column), ↓: decrease, ↑:increase. Bold indicates that values have been calculated by the review authors from data in the study (where applicable, the statistical test used was the independent samples t-test). Blank spaces indicate that there was insufficient data available to answer the question

**Table 6:** Comparison of average testicular volume in 3 studies that compared men with classic testicular microlithiasis (≥ 5 microcalcifications per sonographic image) to those with limited testicular microlithiasis (< 5 microcalcifications per sonographic image)

Authors and reference number	Year	Number in Sample		Testicular volume	
		CTM	LTM	Volume compared to LTM	Statistically significant difference?
***Infertile Population:***					
D’Andrea et al. [9]	2021	46	47	↓	✓
Rassam et al. [32]	2020	53	134	↓	✗
***Population not specified:***					
Xu et al. [35]	2014	97	62	↓	✓

Summary table of the included studies that compared testicular volume in men with classic testicular microlithiasis compared to men with limited testicular microlithiasis. The table indicates the direction of the difference in the classic testicular microlithiasis group compared to the limited testicular microlithiasis group and if the difference was statistically significant. *CTM* classic testicular microlithiasis, *LTM* limited testicular microlithiasis, ✓: Yes (in response to the question in the respective column), ✗: No (in response to the question in the respective column), ↓: decrease, ↑: increase. Blank spaces indicate that there was insufficient data available to answer the question

## Discussion

### Interpretation of results

The results from this review suggest that TM is associated with decreased semen parameters, in particular decreased sperm concentration. As seen in Table 1, 6/6 (100%) results showed lower sperm concentration in the group with TM compared to controls. Of these results, 66.7% were statistically significant. The 2 largest studies in this review by Anvari Aria et al. [51](asymptomatic/healthy population) and Rassam et al. [32] (infertile population), which had a total of 4850 and 2914 participants respectively, contributed to these findings with both studies finding a significant difference in sperm concentration in men with TM compared to men without. Although not included in the results of this review due to WHO semen analysis guideline adherence not being stated, Hiramatsu et al. [33] found that sperm concentration was negatively correlated (*p* < 0.05) with the number of calcifications present. This was also suggested in the comparison of results between participants with CTM and LTM (Table 2).

Although it should be noted that CTM and LTM classifications are not commonly used in clinical practice, they are often included in semen analysis studies. These classifications may indicate how the extent of microlithiasis is associated with semen parameter results. All 6 (100%) studies that compared sperm concentration in CTM and LTM group showed a lower sperm concentration in the CTM group although, only in 2 (33.3%) studies were the results statistically significant. Interestingly, in the study by Anvari Aria et al. [51] that reported semen parameters based on the lateralisation of TM, only the group with bilateral TM showed a significantly lower sperm concentration compared to the group without TM. This may suggest an overall trend of reduction in sperm concentration in those with more extensive disease and is in support of the statement by Xu et al. [35] that ‘the extent of microlithiasis correlates inversely with semen parameters’.

These findings are consistent with the pathophysiology of TM described in the literature as microcalcifications can obstruct seminiferous tubules [28] leading to a lower sperm concentration. Additionally, TM has been proposed as a possible symptom of testicular dysgenesis syndrome (TDS) [52]. TDS was first described by Skakkebaek et al. [53] in 2001 in response to trends of declining male reproductive function reported in the literature [53]. Although the theory has some critics [54],TDS links multiple factors, including Sertoli cell dysfunction, and suggests they are all symptoms of one underlying syndrome that have common pathogenic links [53]. As sertoli cell dysfunction is also proposed as being involved in the pathogenesis of TM [15], this provides a theoretically plausible link between the two conditions. The potential impairment of spermatogenesis as a result of dysfunctional Sertoli cells may also link TM with infertility [55]. Jiang et al. [48] also suggested that TM may affect the blood supply of the testes, thereby impacting spermatogenesis. This could be another explanation for the results seen in this review. However, this hypothesis has been questioned by Mahafza et al. [31] as doppler flow studies for testicular blood vessels were found to be within normal ranges in TM patients included in their study (although, this investigation does not give information on the microvasculature).

Sperm motility in males with TM showed a similar pattern to sperm concentration with 4/6 (66.7%) studies showing lower levels of sperm motility in men with TM compared to participants in the control group (Table 1). Out of these results, 3/4 (75%) were statistically significant. When comparing sperm motility in males with CTM and LTM 3/6 (50%) showed lower sperm motility in the CTM group of which 2/3 (66.7%) were statistically significant (Table 2). Sperm morphology was reported less frequently than other parameters in the included studies and had less conclusive findings. Of studies that reported morphology in TM and control groups, 3/5 (60%) showed lower normal morphology in the TM group in comparison with the control group. Only 1 of these 3 results (33.3%) was statistically significant (Table 1). When comparing sperm morphology in males with CTM to those with LTM 1/4 (25%) studies showed lower normal morphology and this result had an insignificant *p*-value (Table 2).

Although most individual study results were not statistically significant, the results of multiple studies showed an overall pattern of elevated FSH and LH, decreased testosterone and decreased testicular volume in males with TM (Tables 3–6). This trend in hormone values and testicular volume could be investigated in future research as there is insufficient evidence in this review to reach any conclusions. However, these findings may suggest sub-optimal functioning of the testes and a higher tendency to testicular failure in men with TM. A case study of a male with TM by Smith et al. [28] found elevated FSH levels although LH and testosterone were within the normal range. Thomas et al. [34] also found elevated FSH in 2 patients with TM. However, they concluded that these results could be expected in a population of infertile men and germ cell failure could be a differential diagnosis instead of TM. Additionally, D’Andrea et al. [9] found testicular volume to be an indicator of CTM and suggested that infertile males with decreased testicular volume should be offered testicular US to screen for CTM. Both conventional and non-conventional semen parameters have been found to be negatively correlated with testicular volume [56] therefore, causality in the context of TM and its association with semen parameters should be scrutinised in future research. On the whole, there was variability within the results of the secondary outcomes and less data on which to base conclusions on. Further investigation is needed.

### Limitations

There are limitations with the literature included in this review due to heterogeneity in the methodology between studies. The main difference was in the populations that participants were sampled from. Some studies included participants that were from an infertile population, some participants came from an asymptomatic population, and some studies did not specify the nature of the population that that participants were sampled from. Although the potential presence of conditions that act as confounding factors in infertile populations may bias results, the study by Mahafza et al. [31] (one that did not have exclusion criteria for confounding variables for infertility) discovered that even after searching for other potential causes of reduced semen quality, a subset of men in their TM group had decreased semen parameters with no other explanation that could be found except TM. Additionally, heterogeneity in the reporting of results meant that a statistical test of association could not be performed.

Furthermore, the publication year of studies included in this review ranges from 2000–2021 and the quality of ultrasound images between studies may be variable. Steps should be taken to standardise the use of scrotal US in the diagnosis and characterisation of TM in studies investigating semen parameters. The European Academy of Andrology has promoted the standardisation of scrotal US through the “EAA ultrasound study” [57]. Studies also varied in the amount of detail with which they described the methodology behind semen collection and analysis. Future studies into TM and semen parameters should utilise the checklist by Björndahl et al. [58] to encourage higher-quality studies. The use of the NOS to assess the quality of included papers highlighted that sample size calculations were not specified in the papers included in this review and that details of non-respondents were not available. This is likely to be due to the retrospective nature of many of the studies included. Studies were also marked down for a lack of control over confounding variables.

### Implications

Although semen analysis is an important investigation in the evaluation of male infertility, the results from this review should be interpreted with caution. Data on semen parameters does not necessarily equate to clinical outcomes and semen analysis alone is not an indicator of fertility [59]. Determining the clinical significance of decreased sperm values in those with TM would require data on pregnancy rates and live birth outcomes in males with TM compared with a control group without TM and this data is limited in the literature.

Comparison of semen parameters with the WHO 2021 “decision limits”[60] has its limitations [61] however, in this review only 2/9 (22.2%) studies that included sperm concentration values had values on or below the WHO decision limits [60]. Again, this should be interpreted carefully as the WHO decision limits are open to criticism [62] and are often misconstrued as demonstrating a distinction between fertile and infertile males when this is not the case [61].

In the study by Rassam et al. [32], the TM group had a sperm concentration of 29.6 ± 20.4 million/ml (mean ± SD) and the control group had a concentration of 54.3 ± 29.7 million/ml (mean ± SD). In comparison, some studies had minimal statistically significant differences between the TM and control group as demonstrated in the study by Anvari Aria et al. [51] where the sperm concentration in the bilateral TM group was 43 (4.3–74) million/ml (median (10-90th percentile)) and 44 (8.3–120) million/ ml (median (10-90th percentile)) in the control group. This demonstrates the variation between studies and the difficulty in determining how these results might manifest clinically.

For these reasons, we are not able to make definitive clinical recommendations and instead recommend that this is a topic that requires further research. The importance of future research is further highlighted in the context of testicular malignancy, as although men with testicular cancer were excluded from this review, TM and concomitant infertility are associated with an increased risk of testicular malignancy [63]. Considering this, until more data is available, patients with extensive disease may wish to have semen analysis. If parameters are low, they should be advised to report for follow-up if experiencing fertility issues. The rationale for this would be that fertility options could be explored and annual screening for testicular cancer commenced [64].

### Future research

The ideal study to answer the review question would sample participants from a general population and have extensive exclusion criteria to guard against confounding variables. Additionally, as some of the studies had small sample sizes, larger numbers of participants would be beneficial. Studies using advanced sperm function tests in TM patients could provide more indications of the clinical significance of TM [59] whilst the clinical outcomes of TM should be assessed with future studies investigating clinical pregnancy rates. Studies should also adopt consistent definitions of TM to aid with future systematic reviews and meta-analyses. Although ethnic differences in the prevalence of TM have been found [22, 23], the ethnicity of participants was rarely reported in the studies included in this review. We suggest that ethnicity data should be included in future studies where available. It has also been suggested that total motile sperm count is a preferential parameter for expressing the severity of male infertility [65], this measure could be included in future research as an additional point of analysis alongside WHO classifications. Finally, future research should aim to provide clinical guidance on which men with TM would benefit from fertility follow-up and what this follow-up should entail.

## Conclusions

TM is a condition characterised by microcalcifications in the testes. The microcalcifications in TM are thought to block seminiferous tubules and theoretically, there is potential for TM to impair spermatogenesis and the normal functioning of the testicle. This review suggests the presence of microcalcifications in the testes is associated with decreased semen parameters, in particular, decreased sperm concentration. There is also evidence to suggest that those with marked calcification may have a worse sperm concentration than those with less extensive TM. Overall, this review suggests TM may be a risk factor for decreased semen quality. However, the statistical significance of results included in this review should not be conflated with clinical relevance. In the case of TM and semen parameters, clinical outcomes require further investigation, and this review highlights the need for future research on this topic.

## Supplementary Information

Supplementary Material 1.

Supplementary Material 2.

Supplementary Material 3.

Supplementary Material 4.

Supplementary Material 5.

## Data Availability

All data generated or analysed during this study are included in this published article and its supplementary information files.

## Abbreviations

CTM: Classic testicular microlithiasis
LTM: Limited testicular microlithiasis
NOS: Newcastle-Ottawa Scale
TM: Testicular microlithiasis
US: Ultrasound
WHO: World Health Organisation
