# Supplementary material for: Is testicular microlithiasis associated with decreased semen parameters? a systematic review
Source: Basic Clin Androl. 2024 Dec 5;34:23. doi: 10.1186/s12610-024-00238-x (PMC11619182; doi:10.1186/s12610-024-00238-x)
Supplement: Supplementary file 1 — Supplementary Material 1. [file 12610_2024_238_MOESM1_ESM.docx]

**Search Strategies**

| 1. | testicular microlithiasis/ |
| --- | --- |
| 2. | (Testic* adj3 (Microlithiasis or calcification or microcalcification)).mp. [mp=title, abstract, heading word, drug trade name, original title, device manufacturer, drug manufacturer, device trade name, keyword heading word, floating subheading word, candidate term word] |
| 3. | 1 or 2 |
| 4. | sperm count/ or semen parameters/ or total motile sperm count/ |
| 5. | spermatozoon motility/ |
| 6. | ((semen or sperm* or seminal) adj3 (count or number or motility or mobility or morphology or concentration or volume or parameters or quality)).mp. [mp=title, abstract, heading word, drug trade name, original title, device manufacturer, drug manufacturer, device trade name, keyword heading word, floating subheading word, candidate term word] |
| 7. | 4 or 5 or 6 |
| 8. | 3 and 7 |

**Embase:**

| 1. | testicular microlithiasis.mp. |
| --- | --- |
| 2. | (Testic* adj3 (Microlithiasis or calcification or microcalcification)).mp. [mp=title, book title, abstract, original title, name of substance word, subject heading word, floating sub-heading word, keyword heading word, organism supplementary concept word, protocol supplementary concept word, rare disease supplementary concept word, unique identifier, synonyms] |
| 3. | 1 or 2 |
| 4. | Sperm Count/ or Semen/ or Spermatozoa/ or Sperm Motility/ or Semen Analysis/ |
| 5. | ((semen or sperm* or seminal) adj3 (count or number or motility or mobility or morphology or concentration or volume or parameters or quality)).mp. [mp=title, book title, abstract, original title, name of substance word, subject heading word, floating sub-heading word, keyword heading word, organism supplementary concept word, protocol supplementary concept word, rare disease supplementary concept word, unique identifier, synonyms] |
| 6. | 4 or 5 |
| 7. | 3 and 6 |

**MEDLINE:**

| 1. | (Testic* NEAR/3 (Microlithiasis or calcification or microcalcification)) |
| --- | --- |
| 2. | ((semen or sperm* or seminal) NEAR/3 (count or number or motility or mobility or morphology or concentration or volume or parameters or quality)) |
| 3. | #1 AND #2 |

**Web Of Science:**

| 1. | ((testic* W/3 ( microlithiasis OR calcification OR microcalcification )) |
| --- | --- |
| 2. | ((semen OR sperm* OR seminal) W/3 (count OR number OR motility OR mobility  OR morphology OR concentration OR volume OR parameters OR quality)) |
| 3. | #1 AND #2 |

**Scopus:**
