# Supplementary material for: Is testicular microlithiasis associated with decreased semen parameters? a systematic review
Source: Basic Clin Androl. 2024 Dec 5;34:23. doi: 10.1186/s12610-024-00238-x (PMC11619182; doi:10.1186/s12610-024-00238-x)
Supplement: Supplementary file 2 — Supplementary Material 2. [file 12610_2024_238_MOESM2_ESM.docx]

**Quality Assessment Scales**

The following scale was used to assess the quality of cross-sectional studies included in this review.

Newcastle-Ottawa Scale adapted for cross-sectional studies

Selection: (Maximum 5 stars)

1) Representativeness of the sample:

a) Truly representative of the average in the target population. * (all subjects or random sampling)

b) Somewhat representative of the average in the target population. * (non-random sampling)

c) Selected group of users.

d) No description of the sampling strategy.

2) Sample size:

a) Justified and satisfactory. *

b) Not justified.

3) Non-respondents:

a) Comparability between respondents and non-respondents characteristics is established, and the response rate is satisfactory. *

b) The response rate is unsatisfactory, or the comparability between respondents and non-respondents is unsatisfactory.

c) No description of the response rate or the characteristics of the responders and the non-responders.

4) Ascertainment of the exposure (risk factor):

a) Validated measurement tool. (ultrasound) **

b) Non-validated measurement tool, but the tool is available or described.*

c) No description of the measurement tool.

Comparability: (Maximum 2 stars)

1) The subjects in different outcome groups are comparable, based on the study design or analysis. Confounding factors are controlled.

a) The study controls for the most important factor (select one- testicular cancer). *

b) The study control for any additional factor (cryptorchidism, hypogonadism, varicocele, other conditions that can cause infertility, exclusion criteria that allows comparability between groups) *

Outcome: (Maximum 3 stars)

1) Assessment of the outcome:

a) Independent blind assessment. **

b) Record linkage. **

c) Self report. *

d) No description.

2) Statistical test:

a) The statistical test used to analyse the data is clearly described and appropriate, and the measurement of the association is presented, including confidence intervals and the probability level (p value). *

b) The statistical test is not appropriate, not described or incomplete.

The following scale was used to assess case control studies:

Newcastle-Ottawa Scale for Case Control Studies

Selection (Maximum 4 stars)

1) Is the case definition adequate?

a) yes, with independent validation *

b) yes, e.g. record linkage or based on self-reports

c) no description

2) Representativeness of the cases

a) consecutive or obviously representative series of cases *

b) potential for selection biases or not stated

3) Selection of Controls

a) community controls *

b) hospital controls

c) no description

4) Definition of Controls

a) no history of disease (endpoint) *

b) no description of source

Comparability (Maximum 2 stars)

1) Comparability of cases and controls on the basis of the design or analysis

a) The study controls for the most important factor (select one- testicular cancer) *

b) study controls for any additional factor (This criteria could be modified to indicate specific control for a second important factor- cryptorchidism, hypogonadism, varicocele, other conditions that can cause infertility, exclusion criteria that allows comparability between groups) *

Exposure (Maximum 3 stars)

1) Ascertainment of exposure

a) secure record (e.g. surgical records) *

b) structured interview where blind to case/control status *

c) interview not blinded to case/control status

d) written self-report or medical record only

e) no description

2) Same method of ascertainment for cases and controls

a) yes *

b) no

3) Non-Response rate

a) same rate for both groups *

b) non respondents described

c) rate different and no designation
