# Supplementary material for: Is testicular microlithiasis associated with decreased semen parameters? a systematic review
Source: Basic Clin Androl. 2024 Dec 5;34:23. doi: 10.1186/s12610-024-00238-x (PMC11619182; doi:10.1186/s12610-024-00238-x)
Supplement: Supplementary file 3 — Supplementary Material 3. [file 12610_2024_238_MOESM3_ESM.docx]

| No. | Citation | Notes | Reason for exclusion |
| --- | --- | --- | --- |
| 1 | Takacs T, Gulacsi A, Riesz P, Kopa ZS. Clinical relevance of testicular microlithiasis. European Urology, Supplements. 2013;12(4):e1187. | Conference proceeding- newer conference proceeding available | Population crossover |
| 2 | De Santi B, Spaggiari G, Granata ARM, Romeo M, Molinari F, Simoni M, et al. From subjective to objective: A pilot study on testicular radiomics analysis as a measure of gonadal function. Andrology. 2022;10(3):505-17 | Study does not include data on sperm parameters in males with TM. | Wrong outcome |
| 3 | Tarsitano MG, Kanakis GA, Minnetti M, Pozza C, Tenuta M, Sesti F, et al. How reliable is testicular ultrasound in predicting infertility? Proposal of a new grading system. Andrology. 2018;6(Supplement 2):64. | Study does not include data on sperm parameters in males with TM. | Wrong outcome |
| 4 | Liu L, Xia JK, Zhu ZX, Chen WT, Xu Q. Preliminary study of virtual touch tissue imaging quantification in diffuse testicular diseases of male infertility. Acta Histochemica. 2022;124(2). | Study does not include data on sperm parameters in males with TM. | Wrong outcome |
| 5 | Aria SA, Jorgensen N, Nordstrom Joensen U. Prevalence of testicular microlithiasis in 4850 men from the general Danish population and correlation with Semen parameters and male infertility. European Urology, Supplements. 2017;16(13):e3010. | Conference abstract of an included study. | Repeat data |
| 6 | Zhang QH, Lu GS, Shen XC, Zhou ZS, Fang Q, Zhang X, et al. Nanobacteria may be linked to testicular microlithiasis in infertility. J Androl. 2010;31(2):121-5. | WHO adherence not stated (semen analysis) | WHO guideline adherence not stated |
| 7 | Niederberger C. Re: Clinical and Seminal Parameters Associated with Testicular Microlithiasis and Its Severity in Males from Infertile Couples. Journal of Urology. 2022;207(1):212- | Reference searched- full text not found due to reference being incorrect. Paper then excluded due to it being a discussion of another paper included. | Repeat data |
| 8 | Taha EA, Algahny Algahlan HA, Zidan M, Abdelhafez A, Farag FF. Scrotal ultrasonographic findings in obese infertile patients and their correlations to semen and hormonal profile. Turkish Journal of Urology. 2019;45(1):7-11. | No sperm parameters specifically for participants with TM | Wrong population |
| 9 | Raveendran AVO, Ismail SM, Sajeeth Kumar KG. “Snowstorm” testis. Bangladesh Journal of Medical Science. 2021;20(1):194-6. | Case report of 1 patient | Wrong publication type |
| 10 | Takacs T, Rosta V, Kopa Z. Testicular microlithiasis-correlation with testicular dysgenesis syndrome and fertility parameters in infertile couples. Andrology. 2018;6(Supplement 2):84. | Conference proceeding- WHO adherence not stated (semen analysis) | WHO guideline adherence not stated |
| 11 | Qublan HS, Al-Okoor K, Al-Ghoweri AS, Abu-Qamar A. Sonographic spectrum of scrotal abnormalities in infertile men. Journal of Clinical Ultrasound. 2007;35(8):437-41. | No sperm parameters specifically for participants with TM | Wrong population |
| 12 | Rosta V, Takacs T, Kopa Z. Testicular microlithiasis - A link to testicular dysgenesis syndrome and testicular cancer. Andrology. 2018;6(Supplement 2):69-70. | Study has a focus on testicular cancer. | Wrong population |
| 13 | Catanzariti F, Cantoro U, Lacetera V, Muzzonigro G, Polito M. Testicular microlithiasis and dyspermia: Is there any correlation? Archivio Italiano di Urologia e Andrologia. 2014;86(1):20-2. | WHO adherence not stated (semen analysis) | WHO guideline adherence not stated |
| 14 | Takacs T, Varga B, Riesz P, Kopa Z. Testicular microlithiasis - Correlation with TDS (testicular dysgensis syndrome). Andrology. 2014;2):82. | Conference proceeding- newer conference proceeding available | Population crossover |
| 15 | Lotti F, Frizza F, Balercia G, Barbonetti A, Behre HM, Calogero AE, et al. The European Academy of Andrology (EAA) ultrasound study on healthy, fertile men: Scrotal ultrasound reference ranges and associations with clinical, seminal, and biochemical characteristics. Andrology. 2021;9(2):559-76. | Sperm parameters of males with TM not reported | Wrong population |
| 16 | Xu C, Zhang FF, Yang HL, Ma G, Zhang B, Li KJ, et al. The influence of testicular microlithiasis on the outcomes of in vitro fertilisation in a Chinese Han population. Andrologia. 2017;49(8) | Potential population crossover with previous study, original study more appropriate for answering research question. | Population crossover |
| 17 | Abumelha S, Yap TL, Almashat FA, Raheema A, Christopher N, Garraffa G, et al. Ultrasound scanning in the screening of men with subfertility for testicular malignancy-incidence of abnormalities detected and management. European Urology, Supplements. 2015;14(2):e299-ea. | Population in study included patients with various types of lesions found on ultrasound not just TM. Sperm parameters not reported. | Wrong population |
| 18 | Hiramatsu I, Tsujimura A, Miyoshi M, Ogasa T, Miyoshi Y, Ishikawa K, et al. Prevalence of testicular microlithiasis in healthy newlywed men trying for first-time pregnancy. International Journal of Urology. 2020;27(11):990-5. | WHO adherence not stated (semen analysis) | WHO guideline adherence not stated |
| 19 | Mazzilli F, Delfino M, Imbrogno N, Elia J, Spinosa V, Di Nardo R. Seminal profile of subjects with testicular microlithiasis and testicular calcifications. Fertility and Sterility. 2005;84(1):243-5 | Study does not include data on sperm concentration, motility or morphology. Alternative parameters are reported. | Wrong Outcome |
