# Supplementary material for: Is testicular microlithiasis associated with decreased semen parameters? a systematic review
Source: Basic Clin Androl. 2024 Dec 5;34:23. doi: 10.1186/s12610-024-00238-x (PMC11619182; doi:10.1186/s12610-024-00238-x)
Supplement: Supplementary file 4 — Supplementary Material 4. [file 12610_2024_238_MOESM4_ESM.docx]

**Quality Assessment Tables**

Overview of quality assessment scores from use of the Newcastle-Ottawa Scale adapted for cross-sectional studies.

| **Authors and reference number** | **Year** | **Selection** | | | | **Comparability** | **Outcome** | | **Overall Score /10** |
| --- | --- | --- | --- | --- | --- | --- | --- | --- | --- |
|  |  | Representativeness | Sample size | Non-respondents | Ascertainment of Exposure | Based on design and analysis | Assessment of Outcome | Statistical Test |  |
| Aria et al.^64^ | 2020 | * |  |  | ** | * | ** |  | 6 |
| Jiang et al.^61^ | 2013 | * |  |  | ** |  | ** |  | 5 |
| Ou et al.^59^ | 2007 | * |  |  | ** |  | ** |  | 5 |
| Rassam et al.^41^ | 2020 | * |  |  | ** | ** | ** |  | 7 |
| Sakamoto et al.^60^ | 2006 | * |  |  | ** | * | ** |  | 6 |
| Thomas et al.^43^ | 2000 |  |  |  | ** |  | ** |  | 4 |
| Xu et al.^44^ | 2014 | * |  |  | ** |  | ** |  | 5 |
| Yee et al.^62^ | 2011 | * |  |  | ** |  | ** |  | 5 |

**Green= high quality studies (score 7-9)**

**Orange= medium quality studies (scores 3-6)**

**Red= low quality studies (scores 0-3)**

| **Authors and reference number** | **Year** | **Selection** | | | | **Comparability** | **Exposure** | | | **Overall Score /9** |
| --- | --- | --- | --- | --- | --- | --- | --- | --- | --- | --- |
|  |  | Case definition adequate? | Representativeness | Selection of controls | Definition of Controls | Based on design and analysis | Ascertainment of Exposure | Same method for cases and controls | Non response rate |  |
| D’Andrea et al.^45^ | 2020 | * |  |  | * | ** | * | * |  | 6 |
| Mahafza et al.^40^ | 2016 | * |  |  | * | * | * | * |  | 5 |

Overview of quality assessment scores from use of the Newcastle-Ottawa Scale for case-control studies

**Green= high quality studies (score 7-9)**

**Orange= medium quality studies (scores 3-6)**

**Red= low quality studies (scores 0-3)**
